# Passive Droplet Microfluidic Platform for High-Throughput Screening of Microbial Proteolytic Activity

**DOI:** 10.1021/acs.analchem.4c02979

**Published:** 2024-09-25

**Authors:** Luca Potenza, Lukasz Kozon, Lukasz Drewniak, Tomasz S. Kaminski

**Affiliations:** † Department of Molecular Biology, Institute of Biochemistry, Faculty of Biology, University of Warsaw, Miecznikowa 1, Warsaw 02-096, Poland; ‡ Institute of Physical Chemistry of Polish Academy of Sciences, Kasprzaka 44/52, Warsaw 01-224, Poland; § Department of Environmental Microbiology and Biotechnology, Institute of Microbiology, Faculty of Biology, University of Warsaw, Miecznikowa 1, Warsaw 02-096, Poland

## Abstract

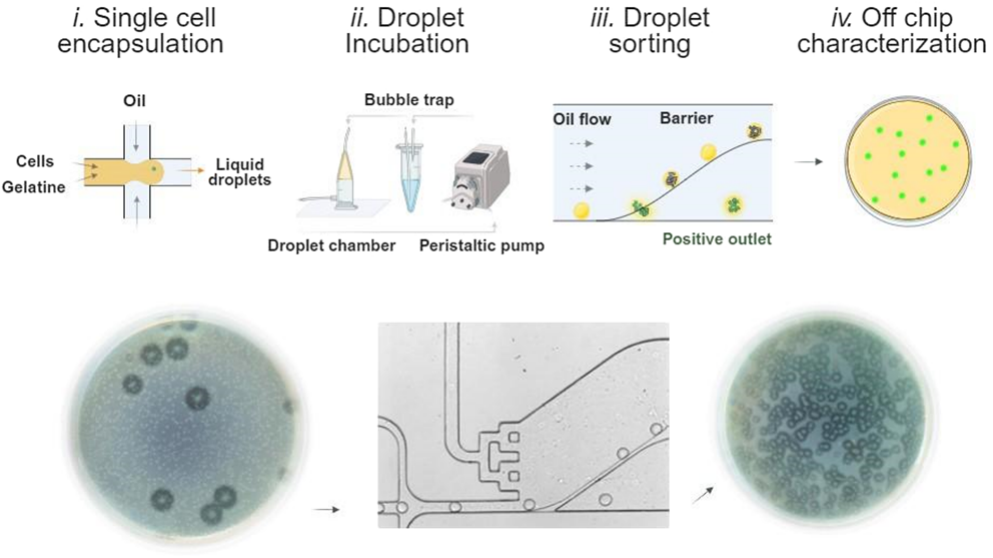

Traditional bacterial isolation methods are often costly, have limited throughput, and may not accurately reflect the true microbial community composition. Consequently, identifying rare or slow-growing taxa becomes challenging. Over the past decade, a new approach has been proposed to replace traditional flasks or multiwell plates with ultrahigh-throughput droplet microfluidic screening assays. In this study, we present a novel passive droplet-based method designed for isolating proteolytic microorganisms, which are crucial in various biotechnology industries. Following the encapsulation of single cells in gelatin microgel compartments and their subsequent clonal cultivation, microcultures are passively sorted at high throughput based on the deformability of droplets. Our novel chip design offers a 50-fold improvement in throughput compared to a previously developed deformability-based droplet sorter. This method expands an array of droplet-based microbial enrichment assays and significantly reduces the time and resources required to isolate proteolytic bacteria strains.

## Introduction

Proteolytic microorganisms play a crucial role in modern biotechnology due to their ability to break down proteins. Proteolytic activity holds a wide range of applications in industries such as bioenergy sector, food processing, detergents and pharmaceuticals production.^1−4^ Proteolytic bacteria also play a role in waste management and biomedical diagnostics. Proteases produced by these strains are widely used as biocatalysts because they facilitate environmentally friendly chemical reactions under mild conditions.^5,6^ In microbiology, the characterization of microbial proteases is based on the isolation of proteolytic microorganisms on selective media.^7^

One of the most common techniques to detect microbial proteolytic activity is cultivation on agar plates containing skimmed milk. Together with lactose, the amino acids from dairy proteins are carbon sources in this agar medium. The proteolytic activity is indicated by a transparent halo around colonies on the milk-white background that appears exclusively around the proteolytic colonies.^8^ Nonetheless, this assay can lead to false positive results, as clear halos could be attributed to lactose utilization and the subsequent production of acid metabolites rather than being caused by the proteases produced by a proteolytic strain.^9^ Moreover, this protocol presents limitations such as low throughput and the necessity for cultivation on a solid medium. Several studies have shown that growing bacteria on agar can negatively affect the recovery of certain microorganisms, thus reducing the overall screening capabilities.^10^

Numerous techniques are employed to quantify protease activity, involving various substrates, both natural and synthetic, and coupled with colorimetric readouts. Common colorimetric assays involve using protease substrates such as azocasein^11^ or chromogenic tripeptides^12^ that are broken down into products that can be detected via absorbance. In the alternative method, the ninhydrin-based method exploits the reaction between ninhydrin and amino acids released during protein breakdown. In this assay, unreacted ninhydrin turns yellow, while amino acid-ninhydrin complexes turn purple. The intensity of the purple color allows for the determination of amino acid concentration resulting from protein degradation.^13^ Despite being widely used, classical protocols for screening microbial proteolytic activity have several limitations. Their sensitivity is too low for accurate detection in cases where proteolytic microbes are present in low quantities, have slow growth rates, or produce proteases with low activity. Even with a transition to a low-volume multiwell plate format, many assays can be costly and time-consuming, which becomes a bottleneck with a large number of samples.

Microfluidic assays offer a distinct advantage by enabling the encapsulation, incubation, analysis, and sorting of individual microbial cultures within controlled microenvironments.^14^ Various studies demonstrated the utility of droplet microfluidics for screening and enriching microbes, for example, based on their growth,^15^ production of antibiotics^16^ or biocatalytic activity.^17^ The detection of proteolytic activity in a droplet-based format has been described only for single mammalian cells, and all these methods are based on fluorescence readouts.^18−20^

In contrast to the above-mentioned protocols, passive methods for microfluidic enrichment of microorganisms leverage the advantages of passive microfluidic sorting. This approach eliminates the necessity for complex instrumentation, thereby simplifying designs, reducing operational expenses, and enhancing user-friendliness. Utilizing phenomena such as surface tension,^21^ viscoelasticity^22^ and the difference in size or density,^23^ passive systems enable selective sorting of droplets. A recent study introduced a system employing passive screening to enrich agarolytic bacteria. The method involved encapsulating microbial cells in microdroplets containing a hydrogel. As the hydrogel gradually degraded, the droplets became more deformable. A double-rail-driven microfluidic design was utilized to passively sort microdroplets. Despite the successful sorting of droplets containing agarolytic microorganisms, the method exhibited a limited throughput, not exceeding a single droplet per second,^24^ which is too low for the enrichment of rare strains from complex microbial consortia.

In this study, we thoroughly explored the utilization of a droplet-based system within the field of microbiology with a specific focus on the selection of proteolytic microbial strains. Key aspects of our research include: i. the design of a passive microfluidic droplet sorter, ii. the utilization of gelatin droplets for the clonal cultivation of microbes, and iii. the establishment of a novel protocol for single-cell studies and microbial isolation. Validation of the efficacy of the deformability-based passive droplet sorter (DPDS) was conducted through an enrichment test to isolate proteolytic strains with exceedingly low abundance within a mock microbial community. Our efforts led to the development of a passive microfluidic workflow demonstrating competitive accuracy and, to some extent, throughput compared to active systems, which typically employ more complex screening methods such as fluorescence-^16^ or absorbance-based^25^ assays.

## Materials and Methods

### Bacteria Cultivation

We used two reference strains: *Pseudomonas aeruginosa* with high proteolytic activity for positive droplets, and*Escherichia coli* DH5α as a nonproteolytic negative control. Single colonies were cultured overnight in LB medium at 30 °C with shaking. Optical density at 600 nm (OD_600_) was measured to assess growth and calculate the cell concentration. The microbial cultures used for cell encapsulation were collected during the exponential growth phase (OD_600_ ∼ 0.5). This optical density was chosen to ensure an accurate calculation of cell occupancy per droplet, as nearly all cells are alive and actively dividing during this logarithmic phase of growth. The droplet cultivation medium was prepared by mixing preheated gelatin (75 g/L final concentration) with LB (0.5x final concentration), and bacteria were gently combined with the gelatin solution to prevent foam formation. A detailed description of the in-bulk cultivation can be found in the Supporting Information section.

### Fabrication of Microfluidic Devices

The microfluidic devices used in this work were designed in AutoCAD (Autodesk), and the master molds were fabricated by standard photolithography and soft lithography. A detailed description of the fabrication process is available in the Supporting Information.

### Gelatin Droplet Generation and Cultivation of Encapsulated Microbes

Bacterial cells from an overnight LB culture were resuspended in rich gelatin medium to the desired concentration. Microemulsions were generated using a flow-focusing droplet generator with an oil phase Novec HFE-7500 (3M) containing 5% 008-FluoroSurfactant (RAN Biotechnologies) at 25 °C. The emulsions were collected in a droplet chamber^26^ and incubated at 40 °C for 96 h. A peristaltic pump supplied oxygen dissolved in the oil to the bacteria inside the droplets while preventing air bubbles from entering. Detailed protocols for droplet generation and microbial cultivation are available in the Supporting Information.

### Passive Droplet Sorting

Glass syringes were attached to PTFE tubing (0.5 mm I.D, 1.0 mm O.D, Bola Bohlender) and locked into an appropriate position on syringe pumps. A first syringe was filled with 1% fluorosurfactant in HFE-7500 oil and used as the first spacing oil. A second syringe was filled with pure oil and was employed as the second spacing oil. A third syringe was filled with pure HFE-7500 oil that was used as a sorting oil. The syringe pumps simultaneously generated flow of oils and droplet emulsion through the device. The flow rates required for sorting proteolytic microcultures within droplets are available in the Supporting Information.

### Determination of Enrichment Factor

Droplets with encapsulated bacterial cells were collected in sterile 1.5 mL Eppendorf tubes. Both the collection tube and the tubing connecting the tube with the microfluidic chip were autoclaved at 121 °C for 15 min before the experiment. Passive sorting of proteolytic microbes was carried out for 30 min, after which the tubing was disconnected from the positive outlet of the sorter and flushed with filtered and sterile HFE-7500 oil. The volume of oil in each sample was adjusted to 250 μL, and the emulsion was disrupted by adding 62.5 μL of 1H,1H,2H,2H-perfluoro-1-octanol (PFO) (Alfa Aesar) and 200 μL of sterile 0.9% NaCl saline solution. The two-phase mixture was first vortexed for 90 s and then centrifuged for 60 s at 2000 rpm to better separate the different phases. The aqueous phase was used to plate the enriched bacteria on Petri dishes containing agar supplemented with 30g/L of skim milk powder. The plates were incubated at 40 °C for 24 h and then examined by visually counting the number of colonies. Colony-forming units were counted for each plate and corresponding dilution, distinguishing between the negative colonies (no transparent halo around the colonies) and the positive colonies (exhibiting a transparent halo).

### Data Analysis

To evaluate droplet sorting in our microfluidic module, slow-motion videos were analyzed using ImageJ. An average background image was generated and subtracted from each video frame to isolate the droplets. The ‘Threshold’ function identified droplet boundaries, and the “Fill Holes” function included droplet interiors. Droplet centroids were extracted using “Set Measurements” and “Analyze Particles”, filtering for moving particles with an area above 8500 pixels^2^. The data were further analyzed in RStudio and Python. Detailed image processing procedures are provided in the Supporting Information.

## Results and Discussion

**Figure 1:**
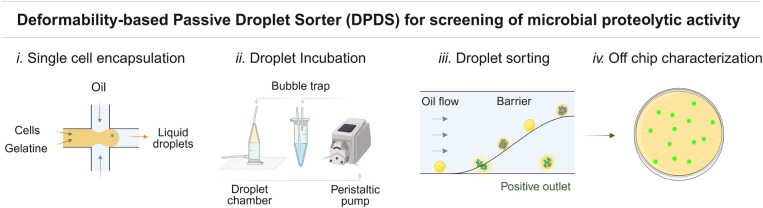
Schematic of the high-throughput passive screening of proteolytic microbial activity. The microfluidic assay is divided into four consecutive steps: i. single bacterial cell encapsulation into liquid gelatin droplets, ii. droplet incubation in dynamic conditions, iii. sorting of droplets based on their deformability properties, iv. off-chip characterization of sorted colonies using standard microbiology methods.

A well-established method for screening microbial proteases in bulk is based on the use of a gelatin-rich medium.^27^ Gelatin, primarily composed of repeating residues of glycine, proline, and hydroxyproline with interspersed arginine, lysine, and aromatic amino acids, serves as a versatile substrate for various proteases. Its composition and structural features make it suitable for degradation by different enzyme classes, including: i. serine proteases (e.g., trypsin), ii. metalloproteases, such as collagenases, iii. aspartic proteases (e.g., pepsin), and iv. cysteine proteases (e.g., papain). Gelatin, a product of the partial hydrolysis of collagen in animal tissues, serves as both a substrate for microbial proteolytic activity and a solidifying agent. Proteases enzymatically break down the gelatin matrix, increasing its solubility by converting proteins into smaller, soluble components. This process disrupts the gelatin structure, reducing its gel-like properties. As a result, when a proteolytic strain is cultivated in gelatin, the medium liquefies. Utilizing the significant change in the deformability properties of gelatin droplets, we propose a microfluidic method for selecting proteolytic microbes comprising four consecutive steps, as illustrated in Figure 1. First, single cells are encapsulated inside picoliter droplets via a flow-focusing device. Droplets are then collected and incubated off-chip inside droplet chambers to allow microbial growth. A constant flow of oil is provided by a peristaltic pump to ensure the oxygenation of the droplets. To prevent the accumulation of air bubbles in the chambers, a bubble trap tube is positioned between the pump and the droplet chamber. Droplets are next reinjected into the deformability-based passive droplet sorter (DPDS) device, where solid droplets (negatives) are pushed along the barrier while more liquid droplets (positives) are squeezed underneath it and collected into the positive outlet. The sorted microbial cultures in droplets are subsequently cultured on a solid selective medium to confirm their proteolytic activity and determine the enrichment factor of the protocol.

The development of the method for selecting proteolytic bacteria involved multiple stages: i. designing and validating a passive sorter which selects droplets based on deformability, ii. defining the parameters for efficient droplet sorting, and iii. optimizing bacterial growth conditions in picoliter droplets and iv. demonstration of the enrichment of proteolytic bacteria from a mock microbial consortium.

### The Design of the Deformability-Based Passive Droplet Sorter

**Figure 2:**
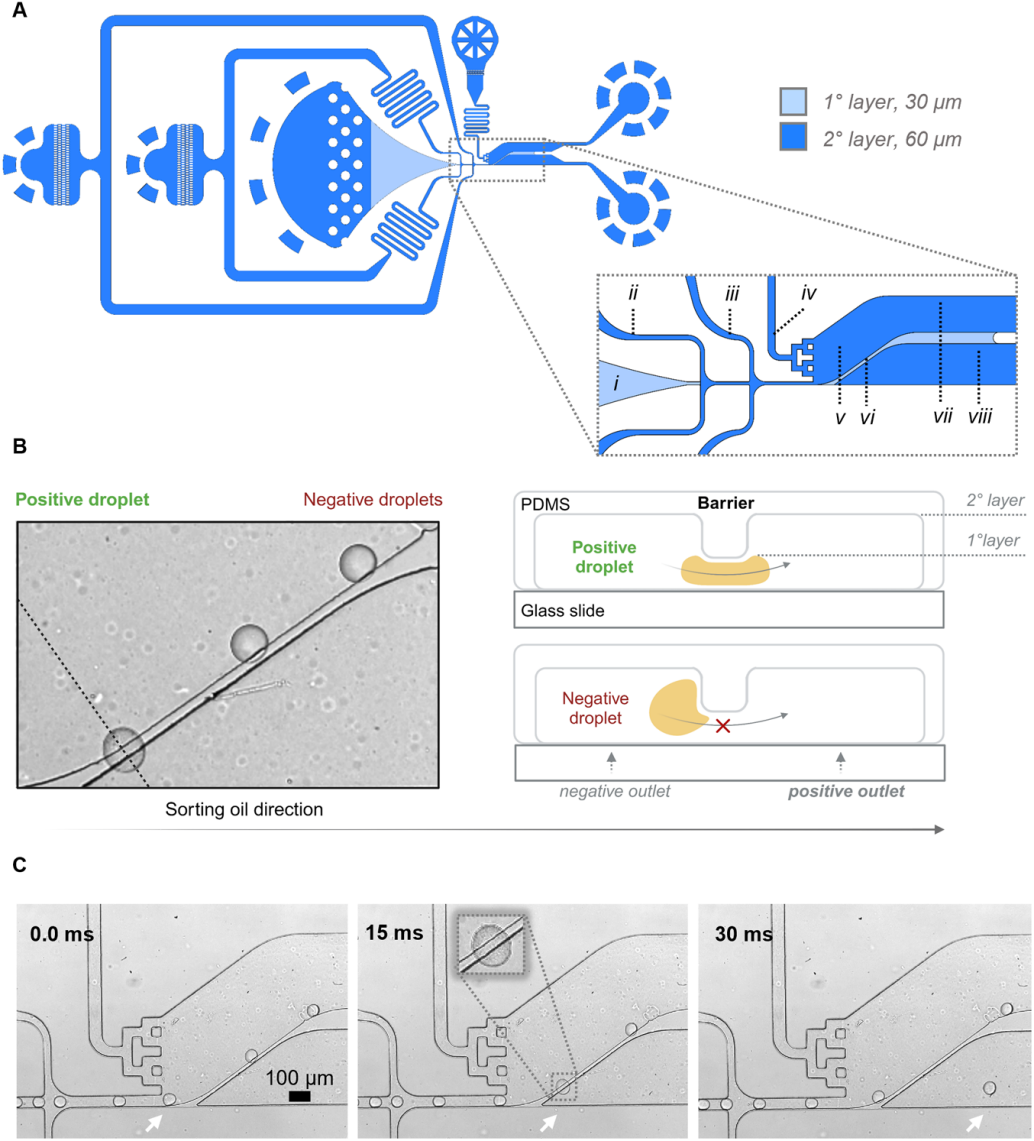
The design of the double-layered DPDS device. Panel A shows the architecture of the passive sorter, which includes the following parts: i. the droplets reinjection chamber, ii., iii. two channels for consecutive streams of spacing oils, iv. the sorting oil channel, v. the main chamber of the chip where sorting is executed, vi. the barrier that derails droplets, vii. the outlet for negative droplets located on the top part of the chamber, and viii. the outlet for positive droplets in the lower part of the chamber. Panel B presents a side view of the DPDS sorting mechanism, illustrating how droplets interact with the barrier. The illustration demonstrates how negative droplets are effectively discarded, while positive droplets pass beneath the barrier for collection in the positive outlet. Microphotographs showing the sorting event of a positive droplet are presented in Panel C.

The layout of the double-layered passive sorter, depicted in Figure 2, represents a novel design that comprises several functional parts. Droplets flowing from the external incubation container first enter the reinjection chamber. The narrower part of the chamber where the second layer of the chip transitions to a part with a height of 30-μm, which forces the droplets to be delivered as a monolayer for efficient spacing with oil. The chamber narrows to a 50-μm-wide main channel and intersects with a reinjection module composed of a double flow-focusing junction delivering two consecutive streams of spacing oil. The scope of such a double-spacing approach is to gradually accelerate droplets, enhancing the overall throughput and simultaneously preventing droplet breakup and/or merging, a phenomenon which we observed for high flow rates of spacing oil and low concentration of gelatin in droplets. Once droplets are evenly spaced, they are directed against a barrier by the sorting oil flowing from the branched tree-like layout. The barrier is 12.5-μm-thick and 30-μm-high structure inclined at 35° to the flow direction. Proteolytic bacteria cultures degrade the gelatin protein matrix and this results in a change of deformability that allow positive droplets to squeeze underneath the barrier and be collected in the positive channel. On the other hand, empty droplets and those with nonproteolytic cultures remain solid, and they are pushed along the barrier to the negative channel and discarded.

### Setting Up Conditions for Generation and Incubation of Gelatin Droplets

The assay we propose requires that solid droplets be effectively derailed by the barrier into the negative channel. As shown in Video S1, an aqueous solution of 7.5% gelatin is first partitioned into monodisperse droplet emulsion (∼1.5–2 kHz, 100 pL) at 25 °C using a flow-focusing junction. Next, these droplets are incubated at 40 °C and subsequently sorted at 20 °C. The room temperature during generation and screening should be controlled by the use of an air conditioning system or the implementation of a microscope environmental chamber. Despite achieving monodisperse droplet generation for a relatively high concentration of gelatin, we encountered droplet merging within 24 h of incubation, which hindered the screening process. To address this challenge, we investigated the impact of static and dynamic droplet incubation on the quality of emulsion.^28^ We monitored the stability of droplets by transferring them from the incubation chamber to a counting chamber to track the emulsion stability. Our findings revealed that dynamic droplet incubation (0.6 mL/h, with 5% fluorosurfactant in HFE-7500) effectively mitigated droplet instability for up to 96 h of incubation. An additional observation from dynamic incubation was the gradual shrinkage of droplets over time–a phenomenon observed before for microbial droplet culture under oil flow.^26^ We determined that droplets volume is reduced by approximately 20 pL after 96 h under cultivation conditions. We accounted for the shrinkage phenomenon by intentionally generating droplets that were initially 100 pL in volume, aiming for an approximate volume of 80 pL on the day of screening.

### Influence of Volume, Gelatin Concentration and Temperature on the Sorting of Proteolytic Cultures

**Figure 3:**
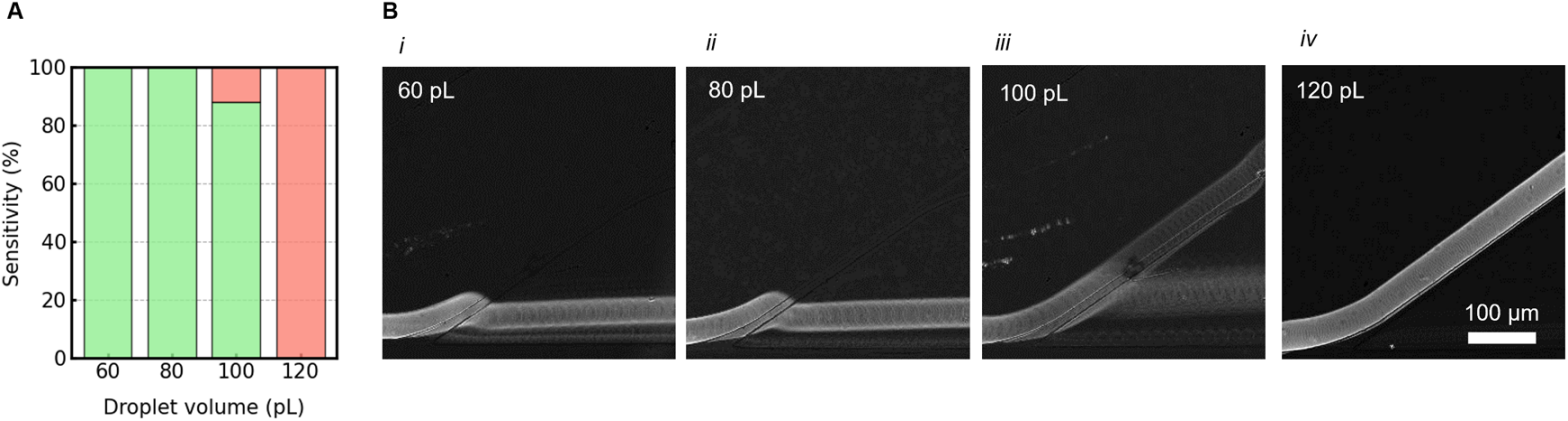
Influence of droplet volume on passive sorting. Panel A presents the impact of droplet volume on the displacement of positive droplets, illustrating results for four different types of LB 0.5x droplets. The column bars plotted demonstrate that the DPDS sorter is highly sensitive to droplet volumes below 100 pL. Green bars represent correctly sorted positive droplets, while red indicates positive droplets directed to the negative channel (false negatives). Panel B showcases sorting accuracy for the different droplet volumes tested. Stack images, based on standard deviations, were generated from videos captured during the passive sorting process. During testing, the sorting oil flow rate ranged from 3 to 4.2 mL/h, and spacing oils were set to 3 ·10^–2^ mL/h and 6 ·10^–2^ mL/h. The room temperature was maintained at 20 °C throughout the experiment.

The development of a barrier-driven sorting mechanism relies on the deformability of positive droplets that can be squeezed and collected in the positive outlet channel. Through our investigations, we have determined three key parameters – volume, gelatin concentration, and temperature – that significantly impact the sorting process. First, the volume of droplets is crucial to the sorting process. In our study, we assessed the relationship between droplet diameter and its effect on barrier-driven sorting within a single DPDS device, while maintaining a room temperature of 20 °C. Droplets of various volumes composed of LB 0.5x medium without gelatin were subjected to deformability-based screening, as illustrated in Figure 3. We found that the optimal volume of droplets for achieving the best performance was 80 pL. Droplet volumes exceeding 100 pL resulted in a high rate of false negatives, as these droplets were too large to deform against the barrier and exit the chip via the positive channel.

Using smaller droplets might enable higher throughput and be advantageous for single-cell assays (e.g., in protein engineering), in which separating growth from enzymatic activity is crucial. However, bacterial growth is more limited in smaller droplets, and the DPDS method, which was optimized for isolating clonal cultures, already accounts for substantial droplet shrinkage during incubation.

**Figure 4:**
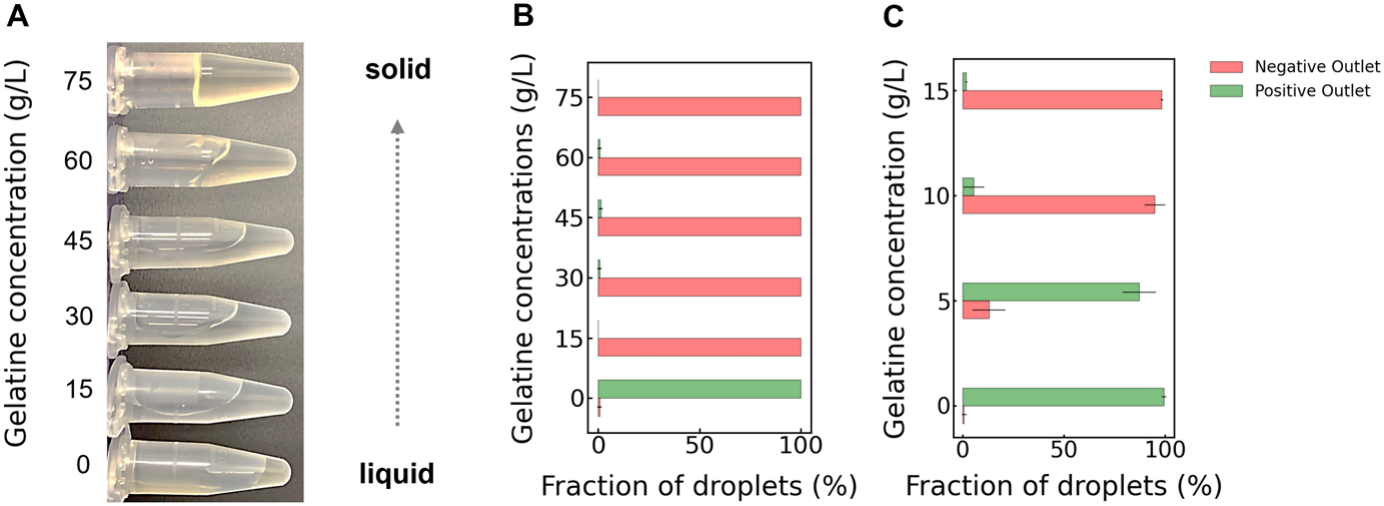
Influence of gelatin concentration and temperature on cultivation medium liquidity. Panel A: The image of various gelatin concentrations captured 30 min after introducing the tubes into the room where the experiment was conducted. The room temperature was maintained constant throughout the experiments using an air conditioning system. The data presented in Panel B, resulting from video analysis, show a high level of accuracy in sorting exclusively the LB 0.5× droplets (no gelatin). The barrier design we developed also proved to be very effective in sorting of droplets containing low amounts of gelatin, as described by the data in Panel C.

Moreover, since gelatin droplets undergo solidification in response to temperature changes, we had to evaluate the influence of gelatin concentrations on the sorting efficacy. We achieved accurate results under optimized temperature conditions, with a switch from 25 °C for droplet generation to 20 °C for 30 min incubation prior to and during the execution of sorting, as illustrated in Videos S6 and S7. We examined six different gelatin concentrations (0, 15, 30, 45, 60, and 75 g/L), as shown in Figure 4, by monitoring the gel/liquid properties of the medium using tube samples placed beside the microfluidic rig. This straightforward experiment allowed us to quickly assess the feasibility of microfluidic screening at a room temperature of 20 °C. The DPDS device completely enriches droplets with 0% gelatin, while discarding all the droplets containing from 1.5% to 7.5% gelatin concentration under optimum flow conditions for maximized throughput. Footages captured during the screening of 0% and 7.5% gelatin droplets, as well as the enrichment of 0% gelatin droplets (10% fraction), are available in Videos S2–S4.

### Microbial Growth Optimization—Influence of Cultivation Medium and Gelatin Concentration

**Figure 5:**
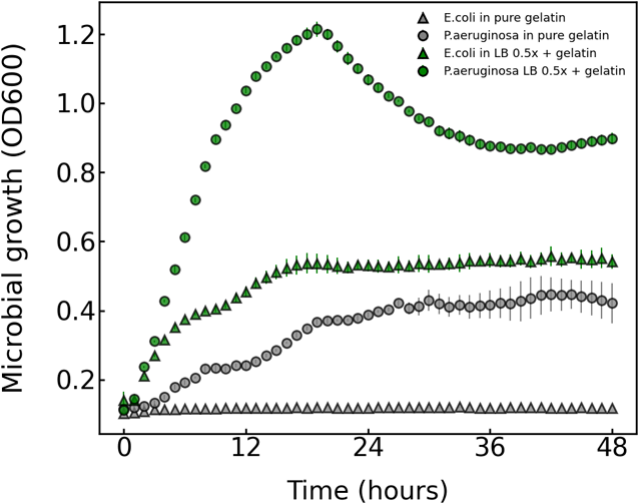
Cultivation medium optimization and growth of reference strains. The microbial growth of reference strains in pure gelatin 7.5% was compared against the medium chosen for in-droplet cultivation – LB 0.5x + 7.5% gelatin. The scatter plot illustrates the growth in bulk of both proteolytic and nonproteolytic bacteria utilized in the development of our microfluidic screening for proteolytic activity. Growth was monitored every hour using a well-plate reader with shaking at 40 °C.

To ensure significant growth of both proteolytic and nonproteolytic strains, we tested various cultivation media, as reported in the Supporting Information section. Our data revealed that 2-fold diluted LB medium (0.5x LB) supplemented by gelatin facilitated faster growth of bacteria both in droplets and in the bulk. Although gelatin serves as a substrate for proteolytic microbes, high concentrations of gelatin unexpectedly inhibited the growth of the proteolytic *P. aeruginosa* strain, as shown in Figure S4. Furthermore, concentrations exceeding 7.5% (w/v) gelatin resulted in emulsion instability, leading to droplet merging during incubation. Consequently, we opted for a final medium composition of LB 0.5x supplemented with 7.5% (w/v) of gelatin, as illustrated in Figure 5. This composition consistently promoted the growth of the reference strains in both bulk and droplet format. Additionally, droplets remained monodisperse during incubation, which facilitated the quantitative screening of proteolytic activity.

Determining the optimal incubation period for microbial replication in droplets was challenging. While *P. aeruginosa* in 7.5% gelatin droplets showed a low rate of false negatives during sorting after 72 h, results from Petri dish and videos indicated that 96 h-long incubation was more effective. This extended incubation time reduces phenotypic variations and enhances the accuracy of proteolytic culture screening using the DPDS device.

Although our study was focused on the proteolytic screening of highly active clonal cultures, evaluating microbial activity at the single-cell level is technically feasible and could provide valuable insights, especially for enzyme screening in single-cell lysates. However, this would require substantial down-scaling of the microfluidic setup. In this study, we optimized the DPDS workflow to select microbes with the highest proteolytic activity. We found that 7.5% gelatin concentration was ideal for forming solid gel beads at 20 °C, significantly reducing false positives. Slow-growing or weakly proteolytic strains can still be detected by either decreasing the gelatin concentration or increasing the incubation time. We determined the minimum gelatin concentration at which droplets are selected as positive, as shown in Figure 4, within a range of very low concentrations (0–1.5%).

### Optimizing Flow Parameters for Efficient Sorting

After several rounds of iterative design and testing, we optimized additional parameters that significantly impact the efficiency of the sorting process. The first tested factors were the droplet volume and the flow rates of both the emulsion and oils used in the sorter system. After several rounds of iterative design and testing, we optimized additional parameters that significantly impact the efficiency of the sorting process. The first tested factors were the droplet volume and the flow rates of both the emulsion and oils used in the sorter system. As a positive reference strain, we used a proteolytic bacterium that was isolated from wastewater sludges and identified as *P. aeruginosa* – as described in the Supporting Information section. Throughout the optimization process and the assessment of sorting accuracy, we employed two types of droplet populations. i. “Negative” - empty ones and droplets with *E. coli* cells – represented a baseline reference. Given the non-proteolytic nature of the *E. coli* cells, such droplets were found to be as negative as the empty ones despite consistent bacteria growth. ii. “Positive” droplets that contained *P. aeruginosa*. To generate microdroplets of uniform volumes, we employed a dedicated flow-focusing device with a 50 × 50 μm junction. Microdroplets were collected within a simple droplet chamber, which facilitates easy reinjection and further manipulation.^28^

**Figure 6:**
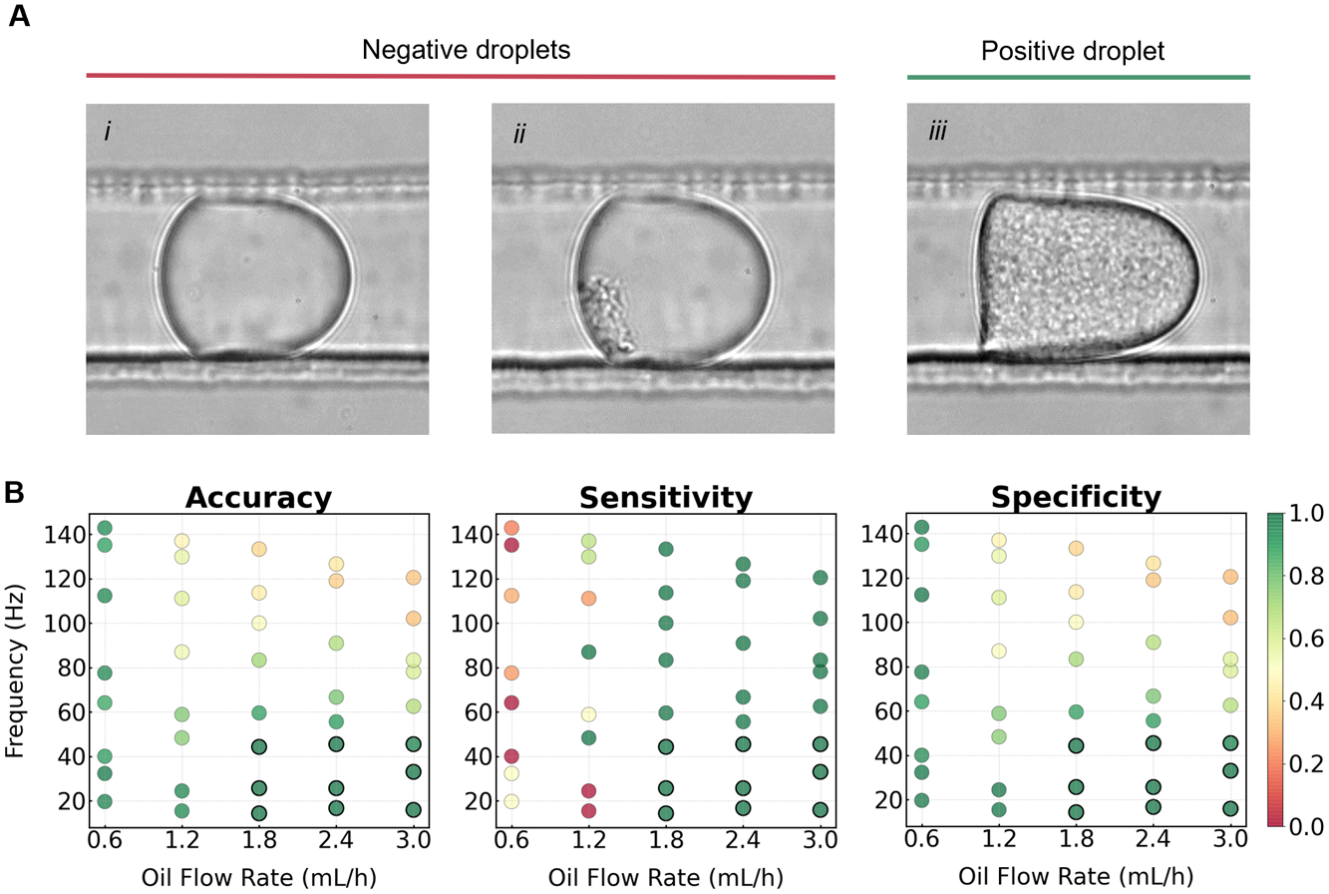
Impact of various flow rates of sorting oil on droplet sorting under microbial cultivation conditions. To mimic the application of the DPDS device toward the screening of an environmental sample, we tested a population of droplets comprising 2% *P. aeruginosa* droplets (positives) and 10% *E. coli* droplets (negatives). In Panel A, we reported the three types of droplets analyzed, i. empty droplet, ii. *E. coli* culture, iii. *P. aeruginosa* culture. We varied the droplet sorting frequencies by adjusting the emulsion flow rate from 20 Hz (using 6 ·10^–3^ mL/h flow rate for emulsion) to 140 Hz (4.8 ·10^–2^ mL/h flow rate for emulsion), and in parallel we tested different sorting oil flow rates (0.6–3 mL/h). The three key parameters that we examined are presented in Panel B: Sorting accuracy, sensitivity, and specificity. The green dots marked with black contours highlight the flow rates of droplets and sorting oil to achieve 100% sorting efficacy.

Next, we proceeded to test different flow rates of sorting oils to determine parameters to achieve optimal sorting accuracy and throughput. The purpose was to determine the numbers of both negative and positive droplets in true and false sorting events. As reported in Figure 6, we detected positive (*P. aeruginosa*) and negative droplets visually, since *E. coli* and *P. aeruginosa* droplets exhibited significantly different colony shapes. We classified droplets containing *P. aeruginosa* (positive) collected in the positive channel as true positives, while empty or *E. coli* droplets (negative) that ended up in the positive channel were deemed false positives. Similarly, the negative droplets in the negative channel were counted as true negatives, whereas positive droplets in the negative channel were identified as false negative incidents. We calculated and employed the following metrics: i. **Accuracy** indicates the combined proportion of the sum of true positive and true negative sorting events relative to the total number of droplets. **Sensitivity**, which represents the true positive sorting events in relation to the total number of collected droplets with proteolytic colonies, and which is calculated as the sum of true positives and false negatives. **Specificity** which measures the true negative events compared to the total number of empty droplets and droplets with*E. coli* (the sum of true negatives and false positives). Throughout this optimization phase, we tested an emulsion composed of a mixture of *P. aeruginosa* droplets (2% of the total), *E. coli* droplets (10%) and empty remaining droplets. We covered a spectrum of droplet flow rates corresponding to various droplet frequencies ranging from 20 to 150 droplets per second. Simultaneously, we explored sorting oil flow rates spanning from 0.6 to 3 mL/h. To maintain consistent spacing between droplets, we kept the spacing oils flow rates constant at 3 ·10^–2^ mL/h and 6 ·10^–2^ mL/h for the first and second spacing oil, respectively. As shown in Figure 6, our results reveal that the best levels of accuracy, selectivity, and sensitivity were accomplished when sorting oil flow rates were within the range of 1.8–3 mL/h and droplet frequencies of up to 50 Hz.

Active droplet sorting systems typically provide a higher level of real-time data acquisition and throughput compared to passive methods like DPDS. For instance, in fluorescence- and absorbance-activated droplet sorting methods, accuracy can be quantified by measuring optical signals.^25,29^ In contrast, passive droplet systems often rely on image detection from videos to characterize sorting efficacy. In our work, maximum efficacy is represented by three parameters: accuracy, sensitivity, and specificity, which ideally should equal 1. After optimization, the DPDS device demonstrated high accuracy and specificity across a wide range of flow rates for sorting oil and emulsions. Sensitivity, a key parameter indicating the number of correctly sorted droplets containing proteolytic colonies (true positives), was also found to be very high for sorting oil flow rates between 1.8 and 3 mL/h at 20 °C. The high level of accuracy was further confirmed in enrichment experiments, where nearly no*E. coli* cells were recovered. Previous microfluidic systems based on hydrogel degradation did not describe performance by using such parameters. For example, a recently published work by Muta et al. used the recovery rate % (target microdroplets successfully sorted from the total detected) and purity % (percentage of target microdroplets in the final population of sorted droplets) as indicators of sorting efficacy.^24^ The microfluidic screening they proposed achieved recovery rates between 71 and 90% and purity levels between 94 and 100%, which represent values lower than those in the parameters presented here.

### Enrichment of Proteolytic Strain *P. aeruginosa* from a Mock Microbial Community Emulating an Environmental Sample

**Figure 7:**
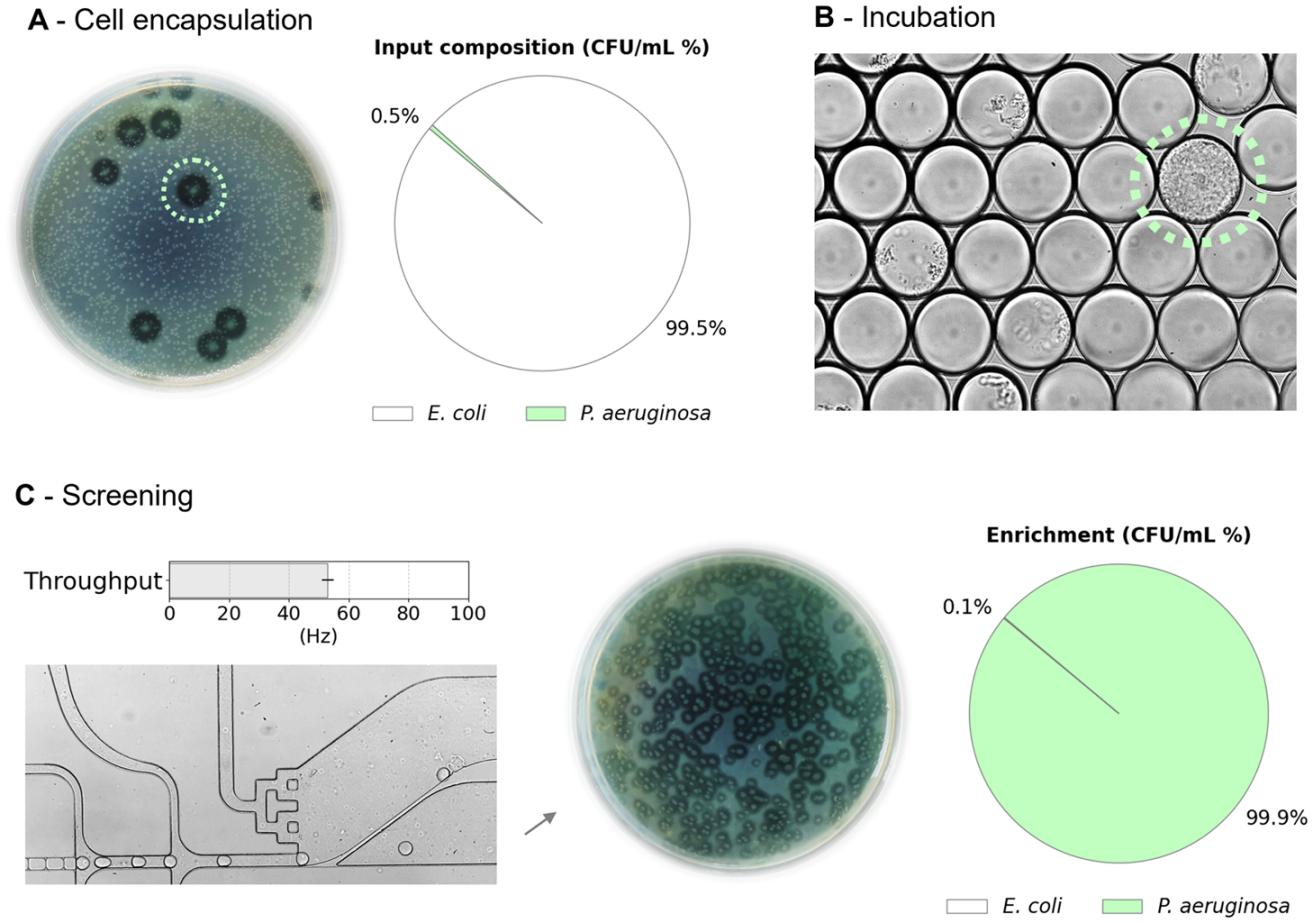
Enrichment of proteolytic strain from the mock 2-strain consortium. Panel A displays a Petri dish containing bacterial colonies present after the initial inoculum in the gelatin medium, prior to the generation of droplets. It predominantly consisted of *E. coli* colonies, with only a few colonies of *P. aeruginosa* indicated by a transparent halo. The relative abundance of *P. aeruginosa*, in terms of CFU/ml, was approximately 0.5% of the total colonies enumerated. Droplets of 100 pL cultivation medium supplemented with 7.5% gelatin were incubated at 40 °C for 72 h - (Panel B), and then screened in the DPDS device as shown in Panel C. The Petri dish in the lower section represented the outlet collection of colonies predominately (>99%) formed by *P. aeruginosa*, distinguishable by the transparent halo around them.

To validate the accuracy of our novel passive sorter, we enriched positive droplets encapsulating single-cell originating colonies of the highly proteolytic *P. aeruginosa* strain from a synthetic microbial consortium as reported in Figure 7. The emulsion primarily consisted of empty droplets (approximately 80%) to minimize the chance of having doublets or triplets of cells per droplet. Analysis of videos from sorting experiments provided insights into the accuracy of the enrichment, including the rate of false-negative and false-positive events. *P. aeruginosa* and *E. coli* were cultured separately overnight in flasks and then mixed to create a mock consortium. This mixture was suspended in an LB medium to achieve a concentration corresponding to approximately λ = 0.2 and encapsulated in 100 pL microdroplets. After overnight cultivation, the emulsion was transferred to the DPDS device, and in 90 min, around ∼270 000 droplets were sorted and collected in sterile tubes. The enrichment experiments were conducted in triplicates. The aqueous phase of the collected and subsequently broken emulsion (positive droplets) was resuspended in sterile NaCl saline solution and then diluted 100 times before plating on agar medium supplemented with skimmed milk. Plates containing at least 500 colonies were chosen to count positive and negative colonies. The presence of active proteases, which can break down the milk substrate, was indicated by halos around positive bacterial colonies.

Although it may seem counterintuitive to plate strains cultivated in droplets, quantitative metrics to evaluate the effectiveness of the DPDS workflow were required. Based on previously described methods,^30,31^ we selected agar plate screening, as both reference strains used in our study exhibit rapid growth on solid and liquid media. Footage captured during the enrichment of this mock consortia is available in Video S5.

The enrichment factors described in Table S2, were determined using previously described methods by Baret et al.^30^ and Zinchenko et al.^31^ The values of factors we calculated were exceptionally high, approximately 4 · 10^5^ and 2 · 10^2^-indicating that our approach effectively enriches proteolytic microorganisms.

## Conclusions

The application of accurate tools to screen microbial consortia holds great promise for the modern microbiology and biotechnology industry.^32^ Today, the development of precise and fast screening of strains is still difficult, but the implementation of droplet microfluidics protocols can facilitate this process. Here, we describe an approach based on single-cell encapsulation and subsequent droplet cultivation for screening microbial proteolytic activity. The enrichment of proteolytic microbes is performed in a novel chip device based on a decreased deformability of gelatin droplets due to proteolytic activity. Ultimately, we identified the optimal conditions that allowed us to passively sort up to 50 droplets per second. To enhance the efficiency of droplet sorting, we conducted a series of experiments to optimize several parameters. These included adjusting the volume of droplets to 100 pL, determining the optimum flow rates for droplets, spacing, and sorting oils as well as identifying the gelatin concentration necessary for generating droplets while maintaining emulsion stability during incubation. Eventually, we achieved a consistent and high level of sorting accuracy, and next we focused on isolating droplets containing proteolytic microbes from a mock consortium that mimics an environmental sample.

In contrast with the current Lab-On-a-Chip formats, the proposed enrichment platform does not require the use of expensive or not commercially available fluorogenic substrates to detect protein degradation. The novel sorter design we introduced relies on a passive sorting mechanism based on the deformability properties of the cultivation medium used to generate picoliter droplets. Compared to other high-throughput methods used for screening proteases via fluorescence-based assays,^20,33^ the simplicity of our method does not foresee the use of electronics or optomechanical equipment, making it more user-friendly. During passive sorting, an operator should focus exclusively on adjusting the combination of oil flows to execute precise sorting and high enrichment. Furthermore, when compared with other passive systems used for deformability-based screening of droplets, our method operates at significantly higher rates, as reported in Table 1. In contrast to the screening of agarolytic activity conducted by Muta et al.,^24^ our device conducted passive droplet sorting at a throughput 2 orders of magnitude higher. The double-rail layout proposed by Muta et al. worked only if droplets flowed at a relatively slow rate to effectively leap to the second rail, which directed them to the positive outlet. In general, rail chips might be less optimal for deformability-based sorting due to the inherent challenge of continuously compressing solid hydrogel droplets during the whole sorting operation, which might lead to clogging of the device as well as droplet collisions and merging.

**Table 1:** Comparison with Other Recently Developed Deformability-Based Sorting of Droplets

Microfluidic Assay	Muta et al.	This work
Target activity	Agarolytic activity	Proteolytic activity
Single-cell encapsulation	yes	yes
Sorter design	double rail	barrier
Enrichment (using mock consortia)	no	yes
Max Throughput (droplet/s)	≤1	∼50
Droplet Volume (pL)	∼65	∼80
Droplet tested (n°)	∼500	∼270 000

The gelatin-based strategy we propose is limited by the different temperatures needed during droplet generation and sorting. This requirement arises from the fact that the deformability of the gelatin medium changes with the temperature. Improvements can involve exploring alternative protease substrates, such as methacrylate gelatin, since this could help address the temperature-related challenges. In future iterations of the DPDS device, single droplets could be deposited into individual wells of plates either by diluting the emulsion of positive droplets^34^ or by using a module for active deposition of single droplets.^35^ Colonies from these deposited droplets could then be scaled up to microliter or milliliter volumes. However, demonstrating these operations is beyond the scope of the current study and will be addressed in further developments of this method.

Our droplet microfluidic protocol, designed for isolating proteolytic microbes, may have broader implications beyond its initial scope. The DPDS system can be used for screening hydrogels to evaluate both their polymerization and degradation processes when exposed to microbial cells. This system is not only rapid but also cost-effective, making it an ideal solution for advancing other studies, such as screening of mutant libraries of*B. subtilis* - a common industrial producer of proteases, or for the directed evolution of enzymes,^33^ as well as detecting the secretion of proteases by single mammalian cells.^20^ Taken together, we believe that the DPDS system could significantly advance both research and practical applications within environmental microbiology. Notably, the DPDS system is ideally suited for monitoring proteolytic bacteria populations and improving our understanding and control of microbial interactions during, e.g., biofuel production and bioremediation processes. Additionally, the system aids in isolating rare microbial strains from diverse environments, which is essential for advancing industrial and environmental applications.

## Supporting Information Available

Detailed protocols of photolithographic microfabrication of molds of microfluidic devices, an image of a profilometer scan of the sorting module, scheme of droplet chamber and incubation strategy, and results of microbial growth optimization (PDF)
High viscosity gelatin droplets generation, Video S1 (MP4)
Passive sorting of 0% gelatin droplets, Video S2 (MP4)
Passive sorting of 7.5% gelatin droplets, Video S3 (MP4)
Passive sorting of 0% gelatin droplets, which make up 10% fraction of emulsion, Video S4 (MP4)
Passive sorting during enrichment of 2-strain mix, Video S5 (MP4)
Influence of 20 °C temperature on droplet sorting, Video S6 (MP4)
Influence of 25 °C temperature on droplet sorting, Video S7 (MP4)
CAD file with designs of microfluidic chips (ZIP)
